# Human Infection with Highly Pathogenic A(H7N7) Avian Influenza Virus, Italy, 2013

**DOI:** 10.3201/eid2010.140512

**Published:** 2014-10

**Authors:** Simona Puzelli, Giada Rossini, Marzia Facchini, Gabriele Vaccari, Livia Di Trani, Angela Di Martino, Paolo Gaibani, Caterina Vocale, Giovanni Cattoli, Michael Bennett, John W. McCauley, Giovanni Rezza, Maria Luisa Moro, Roberto Rangoni, Alba Carola Finarelli, Maria Paola Landini, Maria Rita Castrucci, Isabella Donatelli, the Influenza Task Force

**Affiliations:** Istituto Superiore di Sanità, Rome, Italy (S. Puzelli, M. Facchini, G. Vaccari, L. Di Trani, A. Di Martino, G. Rezza, M.R. Castrucci, I. Donatelli);; St. Orsola University Hospital, Bologna, Italy (G. Rossini, P. Gaibani, C. Vocale, M.P. Landini);; Istituto Zooprofilattico delle Venezie, Padua, Italy (G. Cattoli);; Medical Research Council National Institute for Medical Research, London, UK (M. Bennett, J.W. McCauley);; Emilia-Romagna Region, Bologna (M.L. Moro, R. Rangoni, A.C. Finarelli)

**Keywords:** Influenza, avian influenza A(H7N7) virus, zoonoses, transmission, viruses, Italy

## Abstract

During an influenza A(H7N7) virus outbreak among poultry in Italy during August–September 2013, infection with a highly pathogenic A(H7N7) avian influenza virus was diagnosed for 3 poultry workers with conjunctivitis. Genetic analyses revealed that the viruses from the humans were closely related to those from chickens on affected farms.

In Europe, avian influenza viruses of subtype H7 have been responsible for several disease outbreaks among poultry, which resulted in human infections (*1*,*2*). Notably, since 2000, outbreaks of avian influenza caused by high and low pathogenicity influenza A(H7N1) viruses and low pathogenicity A(H7N3) viruses occurred on poultry farms located mainly in northeastern Italy (*3*). On August 14, 2013, infection caused by a highly pathogenic avian influenza A(H7N7) virus was initially detected on a layer farm in Ostellato, Ferrara Province, Italy, representing the start of an epizootic that affected another 5 poultry farms in Ferrara and Bologna Provinces (Emilia-Romagna Region) during the next 3 weeks. Nearly 1 million chickens on the 6 farms were culled (*4*). All workers (≈200) who participated in depopulating infected premises applied strict infection prevention procedures and were monitored for symptoms. Among the workers, infection with highly pathogenic A(H7N7) avian influenza virus was confirmed for 3 who had conjunctivitis but no respiratory symptoms. We describe the clinical and virologic findings of the investigation conducted with regard to these 3 human cases of influenza A(H7N7) virus infection.

## The Study

On August 28, 2013, a previously healthy 51-year-old poultry worker (patient 1) noted unilateral conjunctivitis. After the worker was examined by a physician, a conjunctival swab sample was collected and tested at St. Orsola Hospital in Bologna; it was positive for influenza A virus subtype H7. Three days later, a 46-year-old poultry worker (patient 2) sought care for bilateral conjunctivitis and other symptoms, such as chills and muscle aches. On September 4, a 49-year-old man (patient 3) sought care for bilateral conjunctivitis. Conjunctival swab samples collected from patients 2 and 3, tested at the same laboratory, also produced positive results for influenza A virus subtype H7.

Patients 1 and 2 worked with breeding and cleaning on a farm in Mordano, Bologna Province; they had not used personal protective equipment (PPE) until August 21, when influenza A(H7N7) virus infection in poultry was diagnosed. Thereafter, they were involved in culling and wore PPE, including face masks with eye protection. Patient 3 had not previously worked with animals, but he participated in depopulation procedures, while wearing PPE, during the 3-week outbreaks on the farms in Ostellato and Mordano. Because the 3 patients had worked for the same poultry company on the affected farms, located inside a 1.5-km–radius area, the date of exposure for each patient is difficult to infer (*4*). All 3 patients were isolated at home; without specific antiviral treatment, symptoms resolved in a few days. Six family contacts were placed under clinical surveillance for 10 days.

Conjunctival swab samples were collected from each patient, and aliquots of these samples were sent to the Istituto Superiore di Sanità, Rome, Italy, where they were confirmed as influenza virus subtype H7N7 by 2 real-time reverse transcription PCR (rRT-PCR) assays (*5*,*6*). A traditional RT-PCR assay was conducted by using specific primers (available upon request), and PCR products were sequenced by using a BigDye Terminator version 3.1 kit (Life Technologies, Austin, TX, USA). Full-genome sequencing was also performed by using the Ion Torrent PGM apparatus (Life Technologies, Carlsbad, CA, USA). Virus isolation in MDCK cells was successful for patient 3 only, whose conjunctival swab sample showed the highest virus load as revealed by a low cycle threshold (18.3) obtained by rRT-PCR (*5*). Full-length gene sequencing was conducted on the clinical sample from patient 3 (GenBank accession nos. KF918334–KF918341), whereas the sequence analysis of the other 2 samples was focused on hemagglutinin and neuraminidase genes and a few regions of the other genes (nt 159–525 and nt 994–1107 of polymerase basic protein [PB] 2, nt 115–453 and nt 1726–2259 of PB1, nt 19–297 of nucleocapsid protein, and nt 715–981 of matrix protein) to trace the avian virus responsible for the human infections. In particular, the virus from the chicken with the index case (A/chicken/Italy/13VIR4527–11/2013) was isolated on August 13 in Ostellato, and 6 other strains were isolated from chickens during the 6 outbreaks that occurred among poultry until September 3, 2013 (*7*) (Table 1). Antigenic characterization of the subtype H7N7 virus isolated from a human was conducted by hemagglutination inhibition assay with turkey erythrocytes (*8*) and H7 reference antiserum. To define virus susceptibility to neuraminidase inhibitors (oseltamivir and zanamivir), we performed the fluorescent MUNANA (2′-[4-methylumbelliferyl]-α-D-N-acetylneuraminic acid)–based assay (*8*).

**Table 1:** Comparison of the nucleotide sequences of highly pathogenic avian influenza A(H7N7) virus isolate from chickens and humans, Italy, August–September 2013*

Virus, location (date)†	PB2					PB1				PA		HA				NP		NA					M	
	232‡	279	333	1044		183‡	1882	2133		1251		471	1018‡	1410		72		515‡	541‡	1040‡	1347		818‡	884‡
Chicken																								
A/CK/4527, Ostellato (Aug 13)§	C	T	T	C		C	C	C		G		A	G	G		G		A	T	C	A		A	G
A/CK/4541, Ostellato (Aug 13)	T	•	•	•		•	•	T		•		G	A	•		•		•	A	•	•		G	•
A/CK/4603, Mordano (Aug 19)	T	•	•	•		•	•	T		•		•	•	•		•		•	A	•	G		G	•
A/CK/4678, Portom (Aug 21)	T	•	•	T		•	•	T		•		•	•	•		A		•	A	•	G		G	•
A/CK/4774, Mordano (Aug 27)	T	•	C	•		•	T	T		•		•	•	A		•		•	A	•	•		•	•
A/CK/5091, Bondeno (Sep 2)	T	C	•	T		T	•	T		•		•	•	•		A		•	A	•	G		G	A
A/CK/5051, Mordano (Sep 3)	T	•	•	–		•	•	T		A		•	•	•		•		•	A	•	•		•	•
Human¶																								
1, Mordano (Aug 29)	T	•	•	•		•	•	T		–		•	•	•		•		•	A	•	–		G	•
2, Mordano (Sep 2)	T	•	•	•		•	•	T		–		•	•	•		•		•	A	•	•		•	•
3, Mordano /Ostellato (Sep 7)	T	•	•	•		•	•	T		A		•	•	•		•		G#	A	A#	•		•	•

*Dots indicate no nucleotide changes; –, notavailable. HA, hemagglutinin; NA, neuraminidase; NP, nucleocapsid protein; M, matrix; PA, polymerase acidic; PB, polymerase basic; Portom, Portomaggiore. †Date indicates date collected. A/CK/4527, A/chicken/Italy/13VIR4527–11/2013; A/CK/4541, A/chicken/Italy/13VIR4541–34/2013; A/CK/4603, A/chicken/Italy/13VIR4603/2013; A/CK/4678, A/chicken/Italy/13VIR4678–1/2013; A/CK/4774, A/chicken/Italy/13VIR4774/2013; A/CK/5091, A/chicken/Italy/13VIR5091–1/2013; A/CK/5051, A/chicken/Italy/13VIR5051–3/2013.  ‡Nonsynonymous substitution. §First isolate from chicken (index case). ¶Clinical samples. #Nucleotide changes found only in the specimen from patient 3 and responsible for the D172G and P347Q amino acid changes.

The 3 patients were infected with an avian-origin influenza A(H7N7) virus. In particular, molecular analyses showed that the hemagglutinin and neuraminidase gene sequences of A/Italy/3/2013 virus isolate (GenBank accession nos. KF712391, KJ136817) were identical to those of the clinical specimens. The hemagglutinin amino acid sequence of this virus, and those from patients 1 and 2, showed complete homology to most of the isolates from chickens, including the hemagglutinin cleavage site containing multiple basic amino acids (PKRRERR*GL) responsible for the highly pathogenic phenotype (*9*). The neuraminidase sequence from the isolate from patient 3 differed from those of clinical specimens from the other 2 patients and most isolates from chickens by 2 amino acids (D172G and P347Q) (Table 1). Neither neuraminidase stalk deletions nor neuraminidase inhibitor sequence-based resistance were detected in the influenza viruses from the humans (*10*,*11*); the drug sensitivity of the viruses was further confirmed by phenotypic neuraminidase inhibitor susceptibility assays performed on A/Italy/3/2013 virus (data not shown).

**Figure 1:**
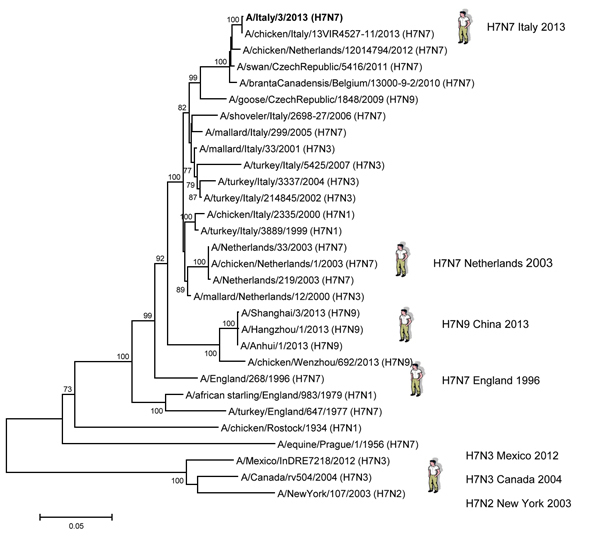
Phylogenetic analysis of the hemagglutinin gene of the influenza A(H7N7) virus, Italy, isolated from humans during August–September 2013. The phylogenetic tree was constructed by using the neighbor-joining method and MEGA 5 software (http://www.megasoftware.net) with 1,000 bootstrap replicates (bootstrap values >70% are shown next to nodes). The influenza A(H7N7) virus isolated from a human in 2013 is shown in boldface. Scale bar indicate nucleotide substitutions per site.

**Figure 2:**
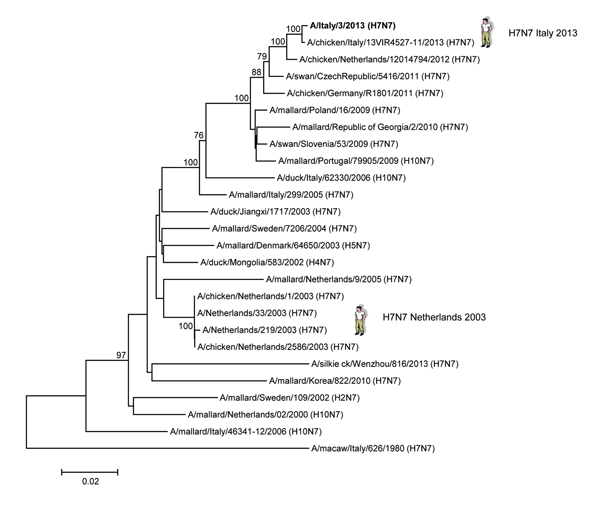
Phylogenetic analysis of the neuraminidase gene of the influenza A(H7N7) virus, Italy, isolated from humans during August–September 2013. The phylogenetic tree was constructed by using the neighbor-joining method and MEGA 5 software (http://www.megasoftware.net) with 1,000 bootstrap replicates (bootstrap values >70% are shown next to nodes). The influenza A(H7N7) virus isolated from a human in 2013 is shown in boldface. Scale bar indicate nucleotide substitution per site.

Phylogenetic analyses of the hemagglutinin and neuraminidase genes (Figures 1, 2) confirmed that the H7N7 isolates from the human patient in Italy were closely related to H7 low pathogenicity avian influenza viruses that had been circulating among wild birds and poultry in Europe during the past 3 years. Antigenic characterization of the A/Italy/3/2013 virus by hemagglutination inhibition assay (Table 2) showed that the virus was recognized poorly by antiserum raised against A/Anhui/1/2013 (H7N9), A/turkey/Italy/214845/2002 (H7N3), and A/turkey/England/647/1977 (H7N7) and recognized well by antiserum raised against A/goose/Czech Republic/1848/2009 (H7N9), A/African starling/England/983/79 (H7N1), and A/turkey/Italy/3889/99 (H7N1).

**Table 2:** Antigenic analyses of influenza A/H7 viruses*

Viruses	Antiserum					
	A/Anhui/ 1/13 (ferret)	A/TK/It/ 3889/99 (ferret)†	A/TK/It/ 214845/02 (ferret)	A/GS/CZ/ 1848/09 (chicken)‡	A/TK/Eng/ 647/77 (chicken)‡	A/AS/Eng/ 983/79 (chicken)‡
Reference						
A/Anhui/1/2013 (H7N9)	**320**	80	80	80	40	80
A/turkey/Italy/3889/1999 (H7N1)	80	**80**	80	40	<40	<40
A/turkey/Italy/214845/2002 (H7N3)	40	80	**80**	<40	<40	40
A/goose/Czech Republic /1848/2009 (H7N9)§	40	40	40	**320**	40	40
A/turkey/England/647/1977 (H7N7)	40	<40	40	40	**160**	40
A/African starling/England/983/1979 (H7N1)§	40	<40	40	40	40	**160**
Test						
A/Italy/3/2013 (H7N7)	40	40	<40	160	40	80

*Data are hemagglutination inhibition titers. Homologous titers of reference viruses to serum samples are indicated in boldface. AS, African starling; GS, goose; TK, turkey. †Antiserum from National Institute for Biologic Standards and Control, UK. ‡Antiserum from Animal Health and Veterinary Laboratories Agency, UK. §Inactivated virus.

Genome sequences of the internal proteins of A/Italy/3/2013 virus and from the clinical samples revealed high identity to the circulating H7N7 strains from chickens in the area. Although deducing the exact exposure for each patient is difficult, partial sequence analysis showed that the virus from patient 1 was more related to A/chicken/Italy/13VIR4603/2013 and that the viruses from patients 2 and 3 were more related to A/chicken/Italy/13VIR5051–3/2013 (Table 1). These 2 viruses from chickens had been isolated during different outbreaks in Mordano (*7*) and differed somewhat from that from the chicken with the index case (A/chicken/Italy/13VIR4527–11/2013). Neither mammalian host adaptation markers, including the E627K mutation in PB2, nor the common mutations associated with adamantane resistance (L26F, V27A/G, A30S/T/V, S31N, G34E) in matrix protein 2 were found in the H7N7 strain from humans (*12*–*14*).

## Conclusions

This study provides further evidence of H7 subtype–specific ocular tropism (*1*). Our molecular findings suggest direct transmission of the virus from chickens to humans; the lack of known host adaptation markers does not support human-to-human transmission. The presence of 2 mutations in neuraminidase from the specimen of patient 3, which contained the highest viral load, might suggest a correlation with the efficiency of infection and replication in the conjunctiva. Indeed, specific neuraminidase mutations have been observed in H7N7 viruses from the Netherlands and have been associated with enhanced replication (*15*). However, further studies are needed to determine their exact role in the pathogenesis of the infection. Clinical surveillance was immediately applied to all exposed workers and cohabiting contacts, and no further human cases of H7N7 infection were identified. For the purpose of investigating human subclinical infections by H7 viruses, a serologic surveillance program is ongoing in the affected areas.
